# Adeno-Associated Viruses (AAV) and Host Immunity – A Race Between the Hare and the Hedgehog

**DOI:** 10.3389/fimmu.2021.753467

**Published:** 2021-10-29

**Authors:** Kleopatra Rapti, Dirk Grimm

**Affiliations:** ^1^ Department of Infectious Diseases/Virology, Medical Faculty, University of Heidelberg, Heidelberg, Germany; ^2^ BioQuant Center, BQ0030, University of Heidelberg, Heidelberg, Germany; ^3^ German Center for Infection Research Deutsches Zentrum für Infektionsforschung (DZIF) and German Center for Cardiovascular Research Deutsches Zentrum für Herz-Kreislauf-Erkrankungen (DZHK), Partner Site Heidelberg, Heidelberg, Germany

**Keywords:** AAV, antibody response, cellular response, capsid, engineering, immune evasion, pre-existing immunity, neutralizing antibodies

## Abstract

Adeno-associated viruses (AAV) have emerged as the lead vector in clinical trials and form the basis for several approved gene therapies for human diseases, mainly owing to their ability to sustain robust and long-term *in vivo* transgene expression, their amenability to genetic engineering of cargo and capsid, as well as their moderate toxicity and immunogenicity. Still, recent reports of fatalities in a clinical trial for a neuromuscular disease, although linked to an exceptionally high vector dose, have raised new caution about the safety of recombinant AAVs. Moreover, concerns linger about the presence of pre-existing anti-AAV antibodies in the human population, which precludes a significant percentage of patients from receiving, and benefitting from, AAV gene therapies. These concerns are exacerbated by observations of cellular immune responses and other adverse events, including detrimental off-target transgene expression in dorsal root ganglia. Here, we provide an update on our knowledge of the immunological and molecular race between AAV (the “hedgehog”) and its human host (the “hare”), together with a compendium of state-of-the-art technologies which provide an advantage to AAV and which, thus, promise safer and more broadly applicable AAV gene therapies in the future.

## 1 Introduction

The hallmark of gene therapy is the delivery of exogenous nucleic acids to cells with the aim to replace missing or defective genes, or to suppress (RNA interference technology) or correct (genome editing) deleterious ones, in order to ultimately ameliorate genetic causes of disease. The ideal delivery vehicle or vector should safely, specifically and efficiently transport the therapeutic cargo and allow expression for the desired duration. Although the delivery of “naked DNA” has progressed all the way to clinical trials, the use of non-viral and viral vectors continues to dominate the field [reviewed in (1–3)]. Viral vectors rely on natural, evolutionary evolved properties of viruses to efficiently evade an organism’s immune surveillance while delivering their cargo to specific cells. Several types of viral vectors are used in gene therapy today, mostly adenoviruses, retroviruses and adeno-associated viruses (AAVs), of which the latter have emerged over the past 20 years as the leading platform for a myriad of applications (1, 2).

AAVs are small, non-enveloped, non-pathogenic viruses endemic in humans and multiple vertebrate species. They belong to the genus *Dependoparvovirus* within the family *Parvoviridae* and are amongst the smallest animal DNA viruses [(4), reviewed in (5–7)]. They carry a ~4.7 kb single-stranded genome that is flanked by two 145 bp ITRs (inverted terminal repeats) forming a characteristic T-shaped hairpin and is packaged in a capsid of T=1 icosahedral symmetry and ~26 nm diameter. Their genome consists of two main open reading frames (ORFs), *rep* and *cap* [reviewed in (7)], and two additional ones encoding the assembly-activating protein (AAP) (8) and the recently discovered membrane-associated accessory protein (MAAP) (9). While the three viral capsid proteins VP1-3 share their C-terminal region, it is VP3, the shortest and most abundant of the three, that determines tissue tropism through receptor binding and interaction with factors in the circulation and interstitial tissue, including but not limited to antibodies [(10–12), reviewed in (2)]. These properties are mainly attributed to nine variable regions (VRI-IX) within VP3 (13).

So far, at least 13 naturally occurring primate serotypes and hundreds of variants have been identified, and countless engineered AAVs with specialized properties are constantly generated [(14–18), reviewed in (1, 2, 19)]. AAVs infect cells by binding to cell surface molecules, identified either as receptors, attachment or viral entry factors, and typically glycans or proteinaceous in nature. Some of these are serotype-specific while others are not, such as AAVR [(20–23), reviewed in (19)]. Binding to these factors is followed by receptor-mediated endocytosis, intracellular trafficking, endosomal escape, nuclear transport, capsid uncoating and finally second-strand genome conversion in the nucleus [reviewed in (19, 24)].

Reasons for the attraction of AAVs as vectors include their broad tropism, low immunogenicity as compared to other vectors, and apathogenicity. Moreover, they are easily engineered as gene delivery vector, by replacing the viral genome with a therapeutic expression cassette, yielding a recombinant AAV (rAAV) with the ITRs as the only essential *cis* elements. Recombinant AAVs transduce cells akin to an infection with their parental wild-type (wt)AAVs, but they cannot integrate into the host cell chromosome in a site-specific manner or integrate at very low frequency, due to the lack of the *rep* gene (25). Still, they can establish long-term transgene expression in both, animals and humans (26–29). Encouraging data in preclinical animal models and in clinical trials [reviewed in (30)] have led to the approval of several gene therapies in recent years, starting with Glybera, a rAAV1 carrying the lipoprotein lipase gene, whose intramuscular delivery aimed at the treatment of lipoprotein lipase deficiency. However, due to the high cost, the scarcity of the disease and the lack of approval in the US, it was withdrawn five years later (2017), despite its therapeutic efficacy (1, 2, 19, 31, 32). The first AAV gene therapy approved by the US Food and Drug Administration (FDA) in 2017 was Luxturna™ or voretigene neparvovec, followed by its approval in Europe end of 2018. Luxturna is an AAV2 vector carrying the *RPE65* gene, which is delivered to the retina to treat an inherited form of blindness caused by a mutation in this gene (1, 2, 19, 31). The second gene therapy approved in the US in 2019 was ZOLGENSMA^®^ (onasemnogene abeparvovec-xioi), i.e., an AAV9 vector carrying the human survival motor neuron (*SMN*) gene and used for the one-time treatment of children under the age of 2 who suffer from spinal muscular atrophy (SMA) (1, 2, 19, 31). Overall, the future of the field is bright, with a 2019 FDA report estimating the yearly approval of 10-20 new cell and gene therapy products by 2025 (2, 33).

Despite the three approved AAV gene therapies and the success of numerous clinical trials with AAVs (1, 2, 30, 31), challenges or impediments remain. This is exemplified by the first clinical trials for the treatment of severe hemophilia B, using an AAV2 vector carrying the gene for coagulation Factor IX (FIX) that was delivered either intramuscularly (34) or infused through the hepatic artery (35). While the first trial was hampered by low-level, short-term expression of <1% of FIX (34), immune responses against the AAV vector were noted in a second trial (35), which were not predicted by any of the preclinical studies in small or large animals (36). No long-lasting systemic toxicity was observed and therapeutic levels of FIX were obtained, but they rapidly declined to background levels, accompanied by a transient increase in liver transaminases. It was later determined that this was due to cytotoxic T-cell (CTL) responses from memory CD8+ T-cells against hepatocytes presenting AAV epitopes *via* major histocompatibility complex (MHC) class I (36–39). In another clinical trial using AAV8 to deliver a codon-optimized *fIX* gene, a short-term supply of immunosuppressants sufficed to block cellular immune responses and enabled long-term expression (27). In additional clinical trials, the presence of pre-existing anti-AAV antibodies governed the efficiency of the gene transfer (40, 41), as will be discussed in more detail below.

The experience and knowledge from these initial clinical trials shaped subsequent efforts to create new generations of AAV vectors that would perform better in humans. In particular, it quickly became evident that immune responses are a major roadblock and require thorough investigation, in order to develop novel, urgently needed strategies to evade or alleviate them. This review will first explore the mechanisms of anti-AAV immune responses and methods to measure them, before focusing on the multifaceted approaches to escape them in a (pre)clinical setting.

## 2 Immune Responses Against AAV

Immunity is the ability of higher organisms to protect themselves from pathogenic invaders, such as viruses or bacteria. Although AAVs are non-pathogenic and vectors derived thereof no longer express any viral proteins, their viral nature renders them a target for the immune system. On top, the fact that AAVs were discovered in human tissues explains why humans carry immunologic memory against them (3, 30, 42, 43).

Generally, the immune system consists of two major arms, the innate and adaptive immunity, which are intertwined and closely regulate one another (44). Adaptive immunity, also called acquired immunity, includes humoral immunity (B-cells, neutralizing antibodies) and cell-mediated immunity (T-lymphocytes, macrophages, natural killer cells).

The following chapters briefly summarize the current knowledge of the role of these arms in anti-AAV immune response in humans before we focus on clinically relevant countermeasures.

### 2.1 Innate Immunity Against AAVs

The innate system is the first line of defense against invaders and, therefore, is relatively non-specific. Professional antigen-presenting cells (APCs), which are present in most tissues, express pattern recognition receptors (PRRs). These recognize structural features on molecules, such as glycans and viral nucleic acids, which are shared between microorganisms and named pathogen-associated molecular patterns (PAMPs) (45). Toll-like receptors (TLRs) are PRRs that are critically involved in immune responses against AAVs together with myeloid differentiation primary response 88 (MyD88), i.e., their universal adaptor [reviewed in detail in (42)]. They are type I transmembrane proteins, which contain leucine-rich repeats and which are located on the cell surface (TLR1, 2, 4-6 and 10) or the endosome (TLR3, 7-9) (46), where they recognize the AAV capsid or the viral nucleic acid (CpG-containing viral genomes and double-stranded (ds)RNA), respectively [reviewed in (42, 45, 47)]. Triggering of PRRs results in nuclear translocation of nuclear factor κB (NF-κB) and interferon regulatory transcription factors (IRFs), which subsequently induce expression of pro-inflammatory cytokines and type I interferons [IFN, reviewed in (3, 42)]. Type I IFNs are the essential link between innate and adaptive immunity [(48–51), reviewed in (3, 47)].

The importance of innate immune responses in AAV gene transfer is subject to intense ongoing investigation. Early studies in mice showed low levels of chemokine induction, at least compared to adenoviral vectors, and the duration was also transient and did not lead to liver necrosis (52). One of the first factors identified to play a role in inhibiting AAV transduction is apolipoprotein B mRNA editing complex 3A (APOBEC3A) (53), a component of the intrinsic immunity. However, the link to innate immunity remains unclear (54). Further studies, mostly performed in the liver, revealed important roles for TLR2 and, most prominently, TLR9 receptors in the early-phase activation of the innate immune system, involving different cell types. TLR2 can sense the capsid of rAAVs (serotypes 2 and 8) on the surface of human non-parenchymal liver cells, such as Kupffer and liver sinusoidal endothelial cells (LSECs). This results in NFκB-mediated immune responses and activation of several interleukins and tumor necrosis factor α (TNFα), but not type I IFN (55). Extensive studies support the role of the viral genome and in particular of the presence of unmethylated CpGs in triggering the innate immune system through the TLR9-MyD88 pathway in different cell types (56, 57), not only in Kupffer cells (58), but also in dendritic cells (DCs) including plasmacytoid (59–61), conventional (59) and monocyte-derived DCs (59, 62). Different TLRs appear to be activated and have distinct effects on different DCs, all of which participate in linking innate and adaptive immunity (45). Still, TLR9 seems to be the most efficient in perpetrating downstream events, such as humoral responses (59) (see below for the link to the adaptive immunity).

Unmethylated CpGs, which are present in the ITRs but also in vector expression cassettes, were shown early on to play a central and enhancing role in the aforementioned TLR9-MyD88 activation (48, 52, 57, 61, 63) [reviewed in (56)]. The detrimental role of CpGs in expression cassettes became evident in clinical trials by the stronger immune responses triggered by codon-optimized transgenes that contained higher CpG levels compared to the wild-type sequences (57), which highlights the importance of the encapsidated nucleic acid. Similarly, the presence of self-complementary rather than single-stranded AAV vector DNA (scAAV vs ssAAV) also results in stronger induction of the innate immune system through TLR9 (58).

In addition to the role of the DNA, the role of double-stranded (ds)RNA produced from AAV vectors has most recently been identified as a factor governing the success of AAV gene therapy (64). Late rather than early innate immune responses are likely to explain the decline in transgene expression that is often observed in patients weeks after AAV delivery. In a recent study, it was hypothesized that the dsRNA produced by the inherent promoter activity of the AAV ITRs was sensed by the PRR melanoma differentiation-associated protein 5 (MDA5). Together with the signaling adaptor mitochondrial antiviral signaling protein (MAVS), MDA5 induces expression of IFN-β, a type I IFN. This induction was seen in different cell lines but, more importantly, also *in vivo* in the humanized liver of mice (64). However, another dsRNA PRR, TLR7, did not show a similar effect (59). Hence, the role and the mechanisms of dsRNA sensing in the innate immune responses require further study (42).

The aforementioned studies of innate immune responses against AAVs yield insights into the connection to the adaptive immunity arm. Indeed, the innate system is considered the key player in the induction of the adaptive responses, which is further corroborated by the fact that transient immune suppression of inflammatory cytokines in clinical trials also suppressed the adaptive immune responses (30). The TLR9-MyD88 pathway is central in this association with both, humoral (51, 59, 62, 65) and cellular immunity (48, 51, 56, 60, 61, 63, 65). In particular, the role of MyD88 in neutralizing antibody induction against the capsid was underlined (51), as well as its role in B-cell induction (59, 65), T helper 1 induction (3, 51, 65), or the shift from Th1 to Th2 (51). In contrast, TLRs are involved in the induction of CD8+ T-cells against the transgene product (51). B-cell induction can also be mediated by cytokines from monocyte-derived DCs (moDCs) (66). The role of TLRs in cell-mediated responses is strongly corroborated by multiple studies. CpG DNA in AAV vectors can induce CD8+ T-cell responses (63), and TLR9 was implicated in capsid antigen presentation through MHCI (51, 60, 63). This process was also shown to require type I IFN (60, 67), next to TLR9, which is secreted by plasmacytoid DCs (pDCs) and binds to its receptor on conventional DCs (cDCs). Subsequently, licensing of the latter activates CD8+ T-cells. Inhibition of this pathway reduced antibody production against the capsid (47, 48). Intramuscular AAV injection, with or without a TLR9 agonist depending on the mouse strain, elicited T-cell responses against the transgene product (59, 62). Similar effects were also observed following systemic administration (68). Finally, at lower doses, AAVs can interact directly with members of the complement system, especially iC3b, which can enhance humoral responses through the classical pathway. Yet, they also bind the complement regulatory protein factor H, which hinders the onset and intensity of antibody formation. At higher doses, AAVs can activate the complement and macrophages, in an antibody-dependent manner (69) [reviewed in (70)].

Together, a wealth of data supports the role of the innate immune system in animals or humans and especially in the induction of adaptive immune responses, as discussed in more detail below.

### 2.2 Adaptive Immunity Against AAVs

Adaptive immune responses follow, and are activated by, the innate system. The adaptive immune system is sophisticated and highly specific to the pathogens. The main actors of the two major branches of adaptive immunity, humoral and cellular, are the B- and T-cells. During development, they produce a vast amount of receptors by rearranging their DNA that recognize the pathogens in an initial encounter. Subsequently, this system generates the so-called immunological memory, which is more robust and is maintained for years after the first invasion (45). The key steps towards immunity include antigen capture and presentation by APCs to lymphocytes, which are in turn activated, clonally expanded and differentiated to effector cells. The effector functions include (1) activation of B-cells and production of antibodies against the pathogen, (2) activation of inflammation, of macrophages as well as of B- and T-cells by helper T-cells, (3) CTL responses to eliminate the pathogen, and (4) induction of regulatory T-lymphocytes to suppress immune responses. The effector phase is followed by contraction of lymphocytes by apoptosis that restores homeostasis and by survival of antigen-specific cells to yield immunologic memory (44, 45).

**Figure 1:**
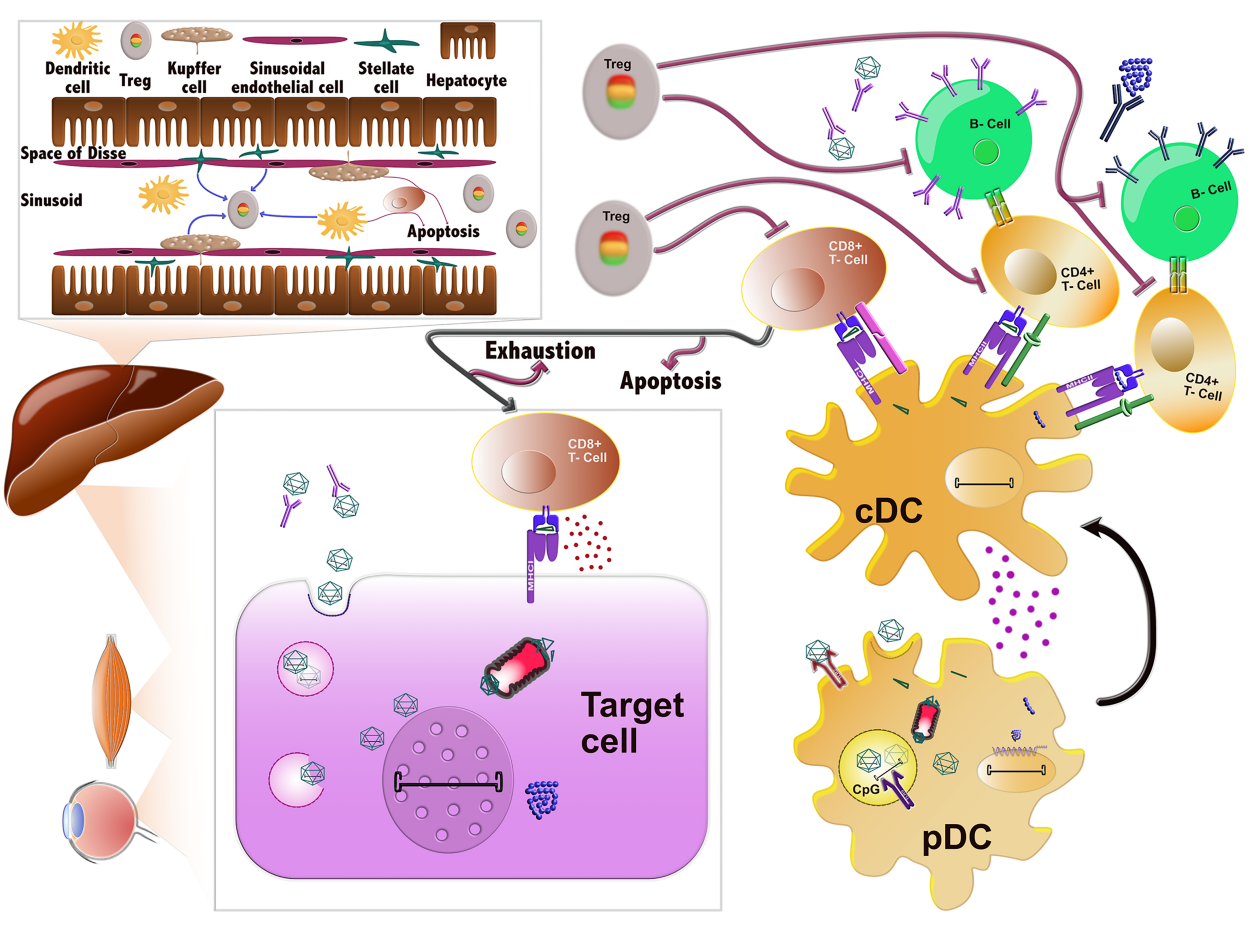
Immune responses against AAVs. AAVs delivered systemically can be neutralized by pre-existing antibodies prior to entering the cells. If they evade nAb binding, they enter the cells through endocytosis and can then be degraded in the endosomes. In certain cell types, such as DCs, their genome or capsid can be sensed by TLR9 and TLR2, respectively, which induces the innate immune response. Alternatively, AAVs can successfully escape the endosome and traffic in the cytoplasm, where they can be ubiquitinated, resulting in capsid degradation. The ensuing peptides can next be loaded to MHC class I molecules and presented on the surface of the cells, which are then targeted and possibly eliminated by CD8+ T-cells (CTL response). After endosomal escape, AAVs can also successfully transduce the cell and deliver their viral genome to the nucleus, where the transgene is expressed. Any misfolded protein encoded by the transgene can be degraded by the proteasome and the ensuing peptides can be loaded onto MHCI and also provoke a CTL response. Sensing of AAV vector components in plasmacytoid DCs (pDCs) by the innate immune system leads to the activation of conventional DCs (cDCs). cDCs employ antigen presentation to cross-prime CD8+ T-cells towards an effector type (Teff) and to activate CD4+ T-cells. The latter can activate B cells, which in turn produce the nAbs against the capsid and transgene product. After prolonged or inadequate stimulation, the CD8+ T-cells can be eliminated by different mechanisms including T-cell exhaustion, anergy or apoptosis. The tolerogenic environment in the liver can also stimulate the production of regulatory T-cells (Tregs), which can suppress the aforementioned immune responses at different stages.

AAV vectors that are presently in clinical evaluation or used as basis for gene therapeutics are typically derived from wild-type AAVs with no or minimal modifications to the capsid, such as peptide insertions or point mutations. As humans are exposed to these viruses early in life, it comes as no surprise that adaptive immunity is a major challenge for gene therapy, as discussed below. Adaptive immune responses against AAVs (overview in **Figure 1**, **Table 1**) have been well documented in clinical trials and investigated in animal models [reviewed in (3, 30, 43, 95, 113)]. The next chapter will explore both, humoral and cellular immune responses against the capsid or the transgene product, as well as methodologies for their detection.

**Table 1:** Summary of immune responses to AAV gene therapy and evasion/prevention strategies.

Response	Strategy to evade them
Pre-existing nAbs against the capsid (35, 71, 72) previous exposure to wtAAVs	Host exclusion from clinical trials (71) route of administration (73), saline flushing (74) immune-privileged organ (75, 76) nAb depletion [plasmapheresis (77), immunoadsorption (78), (IgG)-degrading enzymes (79)]
	Vector novel serotype selection (80) AAV capsid engineering (81, 82) chemical modification of the capsid (83)
Pre-existing nAbs against the transgene previous exposure to recombinant or truncated protein (84)	Host targeting of tolerogenic organs (85)
Activation of the innate system vector capsid (55)/genome (51, 58)/dsRNA (64) sensing CpG containing vector genome (57, 59, 63)	Vector CpG depletion (67) TLR9-inhibitory sequences addition (86) suppression of ITR promoter activity (70)
nAbs against the capsid after gene therapy (79, 87)	Host targeting of tolerogenic organs (88) induction of tolerance (89) B-cell depletion (90)
	Vector vector engineering to avoid antigen presentation (91)
nAbs against the transgene after gene therapy (59, 62, 87) null or missense mutations (92–94) route of administration (92)	Host targeting of tolerogenic organs (37, 95–97) immune suppression (98) B-cell depletion (90)
	Vector tissue-specific expression (promoter, miRs) (99, 100)
Cellular immune responses against the capsid (39, 101) route of administration (102, 103) vector dose (101)	Host immune suppression (27, 98, 101) targeting of immune-privileged organs (102, 103) targeting of tolerogenic organs (104), induction of tolerance (89, 105) cell-type specific expression (promoter, miRs)
	Vector vector selection/engineering to avoid antigen presentation (106–108)
Cellular immune responses against the transgene route of administration (92, 109) vector dose (110)	Host immune suppression (98, 111) targeting of immune-privileged organs (37) targeting of tolerogenic organs (104), induction of tolerance (97) restriction of transgene expression (promoter, miRs) (100)
	Vector vector engineering to avoid antigen presentation (112)
Vector dose (toxicity)	Host lower vector dose capsid/transgene optimization for increased expression

#### 2.2.1 Humoral Immune Responses

Humoral immunity against AAVs, either exhibited by the prevalence of anti-AAV antibodies in the human population (71, 114, 115) but also in animals (116–118), or triggered by AAV vector administration, has been investigated since the early days of AAV vector engineering (119), and it has since been viewed and intensively discussed as a major impediment and exclusion criterion in AAV gene therapy clinical trials. Exacerbating this challenge is that not only up to 90% of individuals in certain areas of the world are seropositive for AAV and that 30-70% are believed to carry neutralizing anti-AAV antibodies (nAbs), but there is also significant cross-reactivity among the known naturally occurring AAV serotypes as well as their synthetic derivatives (115, 120, 121). As the topic of human seroprevalence against AAVs, induced by natural infection or by gene therapy, has already been covered extensively in a flurry of previous reviews including an excellent recent article by Weber in this journal (122) (also references therein), we kindly refer the reader to this literature for more background information. Below, we will instead focus on methodologies for the detection of humoral immune responses against AAVs and then later (chapter 3) discuss experimental strategies to circumvent these.

There are two major *in vitro* methodologies, *i.e*., cell-based assays and ELISAs (enzyme-linked immunosorbent assay), which are used for screening of anti-AAV nAbs and each exhibiting distinctive advantages (123–125). Cell-based assays are more widely used as they are robust and fast. Furthermore, they can distinguish between neutralizing and non-neutralizing, binding antibodies (123, 126). Recently, a variation of these assays has been reported, *i.e.*, a cell-binding assay. This assay, albeit being fast, cannot make this distinction and can only detect neutralization at the level of receptor binding (127). ELISAs, on the other hand, are easy, relatively sensitive and highly useful for determining the immunoglobulin subclasses (114). However, they are typically used to measure binding, not necessarily neutralizing antibodies. There is a high but not absolute degree of correlation between the two assays (121, 128). In cell-based assays, serial dilutions of blood serum or plasma are mixed with equal amounts of an AAV vector, preincubated and then transferred to cells. Transduction efficiency is determined at a distinct time point and expressed as percent to a no-serum/plasma control. The titer is determined as the first dilution at which inhibition exceeds 50% (123, 124, 129, 130), which makes this assay similar to a half-maximal inhibitory concentration (IC_50_) assay. These assays have been performed in numerous variations, using different transgenes (green fluorescent protein (GFP), LacZ and luciferase), cell types (HEK293T or Huh7), serum or plasma, heat-inactivation or not, with adenovirus superinfection or not etc. The sensitivity of the assay was found to decrease with lower cell densities or with GFP [reviewed in (123, 131)]. Of these options, luciferase and HEK293T cells are the most widely used (123, 124, 131), although a need for optimization remains (132). It should also be noted that different AAV purification methods produce different full/empty capsid ratios, which could also impact data transferability efforts (133). Several studies have also attempted to determine the correlation between *in vitro* and *in vivo* assays, but this proved to be challenging and tedious (129, 131, 134). Despite the fact that humoral immunogenicity remains a major impediment for gene therapy in humans and in animal models (3, 30, 47, 116, 135, 136), an international standard assay that takes into consideration key parameters, such as sensitivity and specificity, has yet to be established (135). This underscores the importance of reporting these assays in sufficient detail to allow comparisons between studies and provides the opportunity for a call to the community for additional standardization efforts (78, 122, 137).

#### 2.2.2 Cellular Immune Responses

##### 2.2.2.1 Cellular Immune Responses Against the Capsid

Intriguingly, even though the challenges posed by humoral immunity, predominantly against the AAV capsid, were well established in larger animal models and screened for in patients in clinical trials, the detected cellular immune responses were not as anticipated [reviewed in (3, 30, 43, 137, 138)]. In the first liver-directed clinical trial to treat hemophilia B, hepatic intra-arterial delivery of a recombinant AAV2 vector expressing the human blood-coagulation factor IX resulted in a limited duration of transgene expression and a transient, asymptomatic elevation of liver transaminases in the high-dose group. This was attributed to cellular immune responses towards the capsid that were targeting transduced hepatocytes, as the rise of transaminases was accompanied by a rise in capsid-specific CD8+ T-cells (35). In a subsequent trial using rAAV8 to systemically deliver a self-complementary genome encoding a codon-optimized FIX variant (scAAV2/8-LP1-hFIXco), an asymptomatic increase in serum transaminases or liver-enzyme levels was also observed in the medium- and high-dose groups, together with an increase in capsid-specific CD8+ T-cells in peripheral blood. Still, in patients treated with glucocorticoids, FIX expression was maintained years after vector application (27, 101). It was further established that humans carry capsid-specific T-cells against AAVs (38, 139), and that the elicited immune response is dose-dependent (35, 101). However, immune suppression strategies seem capable of obviating this obstacle, at least to some extent (27, 38, 98, 140–142) [reviewed in (43, 72, 143, 144)]. Besides dose, the route of administration may also play a role in the induction of T-cell immune responses. Intramuscular delivery typically induces stronger cellular responses, although these seem to only account for a reduction of transgene expression, but not for vector elimination (72, 102, 103). They are concomitant with the infiltration of T-cells that do not have a cytolytic (CTLs) but rather a regulatory phenotype, Tregs (145). The latter are formerly known as suppressor T-cells, which are responsible for tolerance to self-antigens. These results once again highlight the complexities of immune responses against AAVs.

The experience gained by the early clinical trials has motivated significant research on the characterization of these immune cells and the mechanisms underlying their induction, stimulation and regulation. APCs are professional, such as DCs, or non-professional, with the former expressing MHC class II molecules and the latter MHCI. Class I MHC molecules, expressed by almost all nucleated cells, mostly present antigens to cytotoxic T-cells, as opposed to MHCII that trigger helper (CD4+) and regulatory T-cells (45). After AAV administration, APCs intracellularly process transgene peptides or proteolytic products through proteasomal degradation of the AAV capsid (146) in the cytosol and cross-present them onto MHCI (60, 147, 148). This, in turn, flags the transduced cells for destruction (39, 149–151). Presentation on MHCII molecules facilitates humoral and cellular immune responses (48, 87, 105). The epitopes on the AAV capsid that are recognized by CD8+ cells are conserved across serotypes (38, 147). These cells are limited in peripheral blood mononuclear cells (PBMCs) (41, 147), which are typically screened during clinical trials, but are more abundant in lymphoid organs, such as the spleen, and recognize epitopes presented *via* major MHCI (147). Capsid-specific T-cells have been found in splenocytes from children (38, 147), which points to the induction of not only humoral, but also cellular immunity early in life after AAV infection and to the maintenance of memory T-cells in secondary lymphoid organs [reviewed in (3, 43)]. These cells express IFNγ, TNFα, IL-2, perforin and the degranulation marker CD107α (66, 147, 152), which equips them with a T-effector phenotype and the ability to exhibit cytolytic activity [reviewed in (3, 43)]. From the aforementioned research, it was long thought that the cellular immune responses against the capsid are mediated by memory CD8+ T-cells generated during childhood after natural infections. Recently, however, the presence of CpG motifs has been linked to the expansion of naïve T-cells directed against epitopes on the capsid. In contrast, memory T-cells react more vigorously to AAV vectors largely depleted of the CpG motifs as well as to empty capsids (63).

Another key aspect is the linkage of humoral to cellular immunity (48, 66). Typically, T-cells are identified by their ability to produce IFNγ upon stimulation with capsid peptides. One notable study that went a step further provided a link between seroprevalence and T-cell reactivity. Seropositive individuals had TNFα-secreting memory CD8+ cells, whereas seronegative individuals showed transient activation not of naïve T-cells, but of natural killer (NK) cells that secrete both, IFNγ and TNFα (66). Additionally, antibody formation requires the CD40-CD40L axis in CD4+ cells (48) and IL-1β and IL-6 in moDCs (66).

Finally, a contribution of the capsid itself in the induction of the cellular immune responses has been reported (87, 153, 154). In more detail, it was shown that AAVrh32.33 can induce stronger humoral and cellular immune responses than AAV8 (87) or other commonly used serotypes. This can be attributed to structural differences and, in particular, to the surface-exposed variable regions, mainly IV (153). This serotype is of particular interest for vaccine applications, due to its low seroprevalence. AAVs as a vaccine have multiple advantages: a single intramuscular application suffices for long-term expression, immunogenicity and protection, and their high thermal stability reduces the thermal-chain requirements, as shown recently for the AAVCOVID vaccine. However, even though large-scale production is feasible, it is challenging to meet the needs of a pandemic (155, 156). The capsid tropism, trafficking and transduction efficiency of APCs also seem to be factors contributing to vector immunogenicity (106–108, 154).

Several studies explored the cellular immune mechanisms in non-human primates (NHPs) and revealed that natural infections with AAVs also produce cellular and humoral responses. Capsid-specific CD4+ and CD8+ cells in rhesus macaques display distinctive differentiation status and function, as well as cell-subset frequencies, with higher proportions of T-effector (Teff) cells as compared to humans (139). Furthermore, unlike chimpanzees, human immune cells do not express CD33-related Siglecs (sialic acid-binding immunoglobulin-type lectins), which are inhibitory signaling molecules thought to downregulate immune cell activation (157).

Cellular immunity against AAV is predominantly evaluated by determining the frequency of capsid-specific T-cells. Two major assays are currently in use, namely, IFNγ enzyme-linked immune absorbent spot (ELISpot) assay and, more recently, flow cytometry combined with intracellular cytokine staining (ICS) (3, 158). ELISpot measures the frequency of T-cells that produce a cytokine, such as IFNγ or, as recently suggested, also TNFα (66), upon stimulation with the appropriate antigen. As a first step, peripheral blood mononuclear cells or splenocytes (usually from humans or from animals, respectively) are isolated, cultured and plated on ELISpot plates that contain membrane bottom wells pre-coated with antibodies against the target cytokine. Afterwards, the cells are stimulated with peptide pools from different AAV capsids. Upon stimulation, the immune cells, granted they have receptors recognizing the antigen, produce cytokines that are captured by the underlying antibodies. Cells are then removed and the cytokine is detected with another antibody, producing spots on the membranes corresponding to each cell that produced cytokines. Use of serial dilutions allows to determine the number of positive cells in a population (139, 147) [reviewed in (3, 113)]. For higher sensitivity, ICS of these immune cells after stimulation with AAV capsid peptide pools can be quantified using flow cytometry (66, 147) [reviewed in (3, 113)], which also allows for multifunctional analysis of T-cells (147). Additionally, because AAV-specific circulating T-cells are rare, an enrichment step based on MHCI tetramers or pentamers and magnetic beads can enhance the detection sensitivity for both assays (41, 66, 147).

As noted initially, the cellular immune responses observed in the first clinical trials, which led to rejection of AAV-transduced cells, were not predicted in any of the small or preclinical animal studies (36). Over time, multiple explanations have been proposed, such as the primate/human source of AAVs, immunological memory in humans, or differences in the immune system [reviewed in (3, 36, 43, 47, 113)]. Extended efforts were then dedicated to develop suitable animal models [reviewed in (113)], including incorporation of a highly immunogenic peptide (SIINFEKL) of ovalbumin in the AAV capsid. To study primary or secondary responses, AAV capsid-specific CD8+ T-cells, derived either from OT-1-transgenic animals (they carry the T-cell receptor for this peptide), or from mice immunized using adenoviral gene transfer of this peptide, are adoptively transferred to recipient mice that are injected with AAVs (60, 63, 159). The adoptive transfer can also be performed without the presence of the peptide or by including an *in vitro* expansion step and further stimulation of the immune system. However, these methods still fail to fully recapitulate the CTL responses [reviewed in (42, 113)].

##### 2.2.2.2 Cellular Immune Responses Against the Transgene Product

Immune responses to the transgene products are influenced by multiple factors, which can be divided into (1) host-specific, such as the underlying mutations (missense, stop codon), the genetic background, disease-related inflammation and pre-existing immunity and (2) vector-specific, such as the AAV capsid and genome, delivery route, tissue-restricted promoters, vector dose, or transgene [reviewed in (30, 42, 95)]. Gene therapy in patients that lack a given protein, due to *e.g.*, a stop codon, is likely to induce a transgene product-specific response. However, underlying mutations (92), even single-amino acid substitutions or transgene-derived cryptic epitopes (84, 93) can induce transgene product-specific cellular responses restricted to tissue-resident T-cells (109). Another determining factor is the genetic background of the patient (37, 94, 160). Disease-specific conditions, such as the dystrophic environment characterized by inflammation, contribute to transgene rejection (161). Pre-existing immunity against the transgene due to protein replacement therapy is also a limiting factor (84). Regarding vector-specific responses, the contribution of the capsid (107, 162, 163) and the vector genome (64, 67, 164) has already been elaborated on in the previous sections. Additionally, the route of administration and promoter-restricted expression are major determinants of immune responses to transgene products. Intravenous delivery results in concurrent expression in the liver and induction of immune tolerance (37, 95), as detailed in the next section. However, intramuscular injection or restriction to this tissue *via* muscle-specific promoters typically results in a stronger immune response (37, 88, 92, 162, 165). This example again highlights the critical role of the promoter in AAV vector constructs and concurrently illustrates the possibilities to reduce immune responses through a meticulous selection of promoters and other regulatory elements. Ideally, this results in the detargeting of vector gene expression from APCs and, thus, avoids presentation of transgene peptides on MHCI and subsequent CTL-mediated clearance of the transduced cells.

Such regulatory elements also comprise binding sites for tissue- or cell-specific mi(cro)RNAs, which can be easily included in the 3’ untranslated region of an AAV vector expression cassette and which will shut down unwanted gene expression in cells expressing the selected miRNA(s). For instance, this strategy has been exploited in the past to purposely detarget AAV transgene expression from the liver, by incorporating binding sites for the liver-specific miR-122 into the recombinant AAV genome (166–168). Most relevant in the context of anti-AAV immune responses are miRNAs that are abundantly expressed in professional APCs, especially miR-142 (169) or, as reported most recently, miR-652-5p (99). As shown repeatedly, inclusion of binding sites for these miRNAs can diminish both, antibody formation and CTL responses, in turn boosting transgene expression and extending its persistence in mice. Impressively, combination of binding sites for miR142 and miR652-5p even enabled robust expression of the highly immunogenic ovalbumin *in vivo*, through detargeting from APCs, inhibition of CTL activation and suppression of Th17 responses (99). The fact that incorporation of miRNA binding sites into AAV vectors is technically simple and that saturation of the endogenous miRNA/RNAi pathway is unlikely (due to the artifical design of these sites that bind miRNAs with perfect complementarity) makes this strategy very appealing and versatile. Finally, vector dose also plays a significant role in defining the T-cell immune response (110) against the capsid and the transgene (84, 170, 171).

Despite the possibility of immune responses against the transgene product, there are few reports of clinical trials encountering this limitation. This could be attributed to residual natural protein expression, to the type of application (*e.g.*, gene replacement therapy), to the preconditions of the individuals, to vector delivery to immune-privileged organs, to the induction of immune tolerance and/or exhaustion, or to the application of immune suppression [reviewed in (3)]. Nonetheless, some transgene product-specific immune responses were observed in vector-treated individuals. In a phase I/II clinical trial, six Duchenne muscular dystrophy patients received the mini-dystrophin transgene intramuscularly. Dystrophin-specific cytolytic CD8+ T-cells were observed in all patients after treatment and in two before (84). T-cell responses were also observed in a separate clinical trial to treat another monogenic disorder, α-1-antitrypsin (AAT) deficiency. Intramuscular delivery resulted in AAT-specific T-cell responses in two participants as well as in a reduction in expression in one of them (172). Finally, in a phase I/II clinical trial to treat mucopolysaccharidosis type IIIB syndrome (Sanfilippo type B syndrome), an rAAV2/5 vector carrying the human α-N-acetylglucosaminidase (NAGLU) was delivered intracerebrally. In three of the four patients, circulating T-cells that produced TNFα upon stimulation with NAGLU peptides were detected but later subsided, indicative of the development of tolerance (173).

#### 2.2.3 Immune Tolerance and Exhaustion

Immune tolerance is a vital part of the immune system, which encompasses a broad spectrum of processes that result in a state of non-reactivity towards antigens or immune homeostasis. This ensures protection from harmful, excessive immune responses inside the host, such as against self-antigens or against chronic infections and the ensuing inflammation that can cause significant tissue damage. The major mediators of immune tolerance are the regulatory T-cells, which include the natural and the induced, also called adaptive, Tregs (nTreg and iTreg), located in the thymus and in the periphery, respectively [reviewed in (143, 174)]. Tregs are the major actors involved in inducing systemic tolerance through liver-directed gene transfer, with the liver being a long recognized tolerogenic organ (175). Different markers are used to identify Tregs, most frequently CD4 and CD25 extracellularly and FoxP3 (forkhead box P3) intracellularly (CD4+CD25+FoxP3+ T-cells) (143, 175). The transcription factor FoxP3 is central to establishing the regulatory lineage. iTregs have a transient expression of FoxP3, whereas it is stable in nTregs (176). Tregs mediate tolerance *via* interaction with CD8+ T-effector cells, whereby they can either inhibit proliferation and IFNγ secretion, or induce cell death through granzyme or perforin (177–179). Tolerance through Tregs is also mediated in the liver draining lymph nodes *via* secretion of immunosuppressive cytokines, such as IL2 or IL10 that cause Teff anergy, exhaustion or suppression (88, 180–182), or differentiation of naïve CD4+CD25- T-cells into Tregs (182) [reviewed in (138)].

Different APCs in the liver are critical for the induction of tolerance (96, 110). Kupffer cells (KCs), *i.e.*, liver-resident macrophages, have a less mature phenotype and can induce expansion of Tregs or the conversion of Teff to Tregs, *via* programmed death ligand-1 (PD-L1) expression and IL-10 production (180, 182, 183). Liver sinusoidal endothelial cells (LSECs) are one of the first liver cells to recruit lymphocytes. Yet, due to high levels of IL-10, priming of T-cells is inefficient, thereby promoting Tregs (175, 184) [reviewed in (3, 95, 143, 175)]. Hepatic DCs also have the capacity to induce and maintain tolerance [reviewed in (175)]. Defective antigen presentation in the liver lymph nodes also results in T-cell exhaustion (110). T-cell exhaustion, that is CD8+ T-cells without effector functions, can be caused by interactions with Tregs, cytokines or by activation of inhibitory receptors, such as PD-1, and can also mediate long-term transgene expression (88, 185, 186). Remarkably, these cells persisted in human muscle biopsies even five years post-vector delivery (185).

## 3 Strategies to Evade Immune Responses Against AAVs

**Figure 2:**
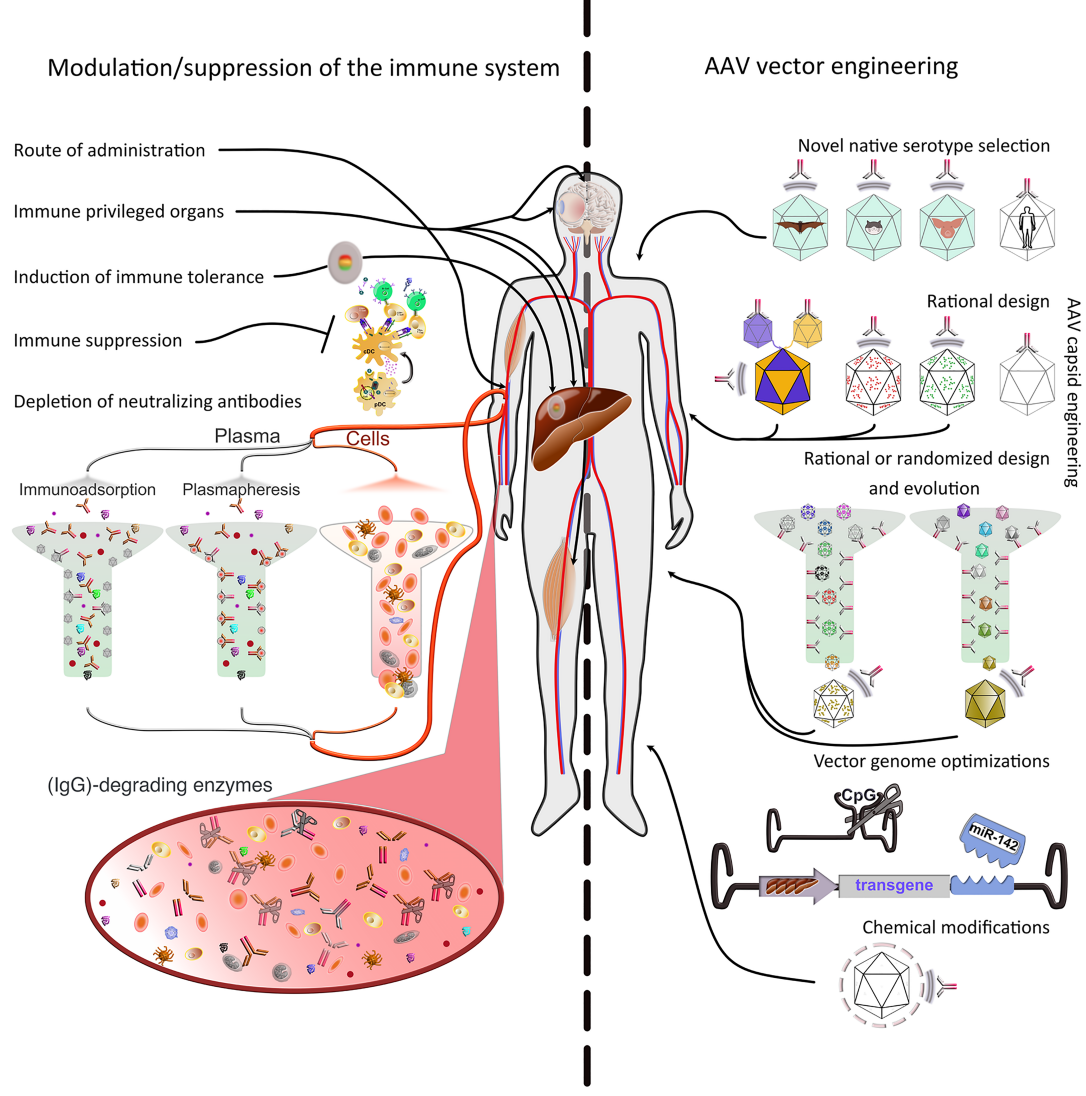
Strategies to evade immune responses. Several approaches to evade immune responses have been applied in the clinic or explored on a basic research level. They can be roughly divided into two main classes, *i.e*., 1) modulation or suppression of the subject’s immune system and 2) AAV vector engineering. 1) Modulation/suppression of the immune system. The route of administration is decisive in evading pre-existing immunity. Targeting immune-privileged organs, when possible, provides protection not only by evading immune responses, but also by inducing regulatory responses. The latter can also be induced through multiple exogenous interventions. Additionally, pharmacological immune suppression has been used extensively in the clinic. Pre-existing immunity (nAbs) is particularly difficult to evade or suppress. Promising approaches include plasmapheresis or the use of immunoadsorption columns *ex vivo* after separation from the cellular parts of the blood. Furthermore, recent studies have used IgG-degrading enzymes *in vivo*. 2) AAV vector engineering. The use of native serotypes from species other than humans or NHPs, or engineering the capsid of existing serotypes (predominantly from primate species) are advantageous strategies to evade immunity. The current serotypes can be modified in defined positions (rational design) based on acquired knowledge about sequence-structure-function relationships. To increase variability, the rational design strategy can be used to generate libraries, which then can be evolved/selected *ex* or *in vivo*. Libraries can also be fully randomized and interrogated for multiple properties, not only immune evasion. Moreover, the viral genome can be optimized to minimize antigen presentation (e.g., *via* cell-type specific promoters, miRNAs to prevent expression in APCs or peptides to inhibit antigen presentation) or immune system induction (CpG depletion). Finally, the capsid can be protected from the immune system by coating with molecules, such as PEG, engulfment in exosomes, or display of immune evasion peptides on its surface.

AAV vectors have been used successfully in numerous clinical trials and in gene therapies. Still, as detailed above, the host immune system poses a substantial barrier to their broad and effective application. A major but unsatisfactory solution thus far has been the identification of patients with pre-existing, typically humoral immunity and their exclusion from participation. Importantly, several additional approaches were also widely explored to include more patients for whom gene therapy might be a preferred, if not the only therapy available. These can be classified into two major categories, *i.e*., modulation/suppression of the immune system or engineering of the AAV vector on the level of capsid and/or transgene (**Figure 2**, **Table 1**).

Below, we will discuss a selection of these approaches that have been published in a large body of work and that range from basic research all the way to the clinic.

### 3.1 Modulation/Suppression of the Immune System

#### 3.1.1 Route of Administration

The route of administration has been strongly implicated in the inhibition by, and the induction of, immune responses. The exact choice is usually determined by the type of disease. Examples for administration routes include intravenous for hemophilia or liver/heart diseases, intramuscular or intravenous for muscle diseases, or intracerebral, intraparenchymal, intrathecal, or intravenous for neurological diseases [reviewed in (1)]. Direct injection into the target organ, such as intramuscular injection (73), is the most straightforward method to avoid circulating antibodies. Additionally, saline flushing to avoid contact with nAbs in combination with direct or balloon catheter-guided vector injection has shown promise (74). Nonetheless, this does not obviate the generation of immune responses after gene therapy (187), which could eliminate transgene expression later on, as explained in detail before. Fortuitously, several delivery methods exist today that facilitate the evasion of these responses (immune-privileged organs) or their manipulation (immune tolerance or T-cell exhaustion).

#### 3.1.2 Immune-Privileged Organs

The intravascular or intravenous route of administration has been predominantly associated with inactivation of AAVs by nAbs in the circulation and interstitial tissues (35, 40, 116, 117, 135, 188). Thus, it is encouraging, albeit not a panacea, that certain tissues provide shelter from the immune system. Three organs are considered immune-privileged, namely, the brain, the eye and the liver.

##### 3.1.2.1 Brain

The CNS, and in particular the brain, have long been considered to be isolated from the immune system by the physical blood-brain barrier (BBB). Moreover, the brain lacks classical draining lymph nodes and APCs in the parenchyma. While these concepts have been challenged (189), CNS gene transfer, either to the cerebrospinal fluid (CSF) (75, 190) and, to a lesser extent, after intraparenchymal injection (191–193), remains comparatively successful notwithstanding the presence of circulating nAbs. Still, in patients with high titers of circulating antibodies, even CNS gene transfer is inhibited (134, 194). This was exemplified in a study in which preimmunization of mice after intramuscular injection hindered subsequent brain delivery. On the other hand, passive transfer of NHP serum containing nAb to mice did not impact gene transfer to the hippocampus or the thalamus (134).

##### 3.1.2.2 Eye

The ocular immune privilege, akin to the one in the brain, was first described in the middle of last century. It is mediated by a blood-retina barrier (195), absence of efferent lymphatics, presence of elevated concentrations of immunomodulatory molecules and immunoinhibitory factors, and finally the ACAID system (anterior chamber-associated immune deviation), which is typically stimulated after perturbation of the ocular integrity. ACAID induction by intraocular antigens results in induction of Tregs, immunomodulators in the aqueous humor, anti-inflammatory cytokines and F4/80 macrophages that present the antigen to clusters of immune cells in the spleen (196, 197). The induction of a deviant immune response has already been reported for adenoviral and AAV gene transfer to the subretinal space (198). In combination with other mitigating factors, such as the low effective dosage and hence minimal toxicity and low manufacturing burden, this well-characterized immune privilege has catapulted ocular gene therapy to the forefront of the field [reviewed in (30)], with multiple clinical trials concluded or underway, culminating in the approval of Luxturna™ (199). There are two major delivery methods, subretinal and intravitreal, of which the latter is slightly more immunogenic (76). Although pre-existing immunity can pose a challenge to intravitreal delivery (200, 201), several clinical trials were successful due to the immune-privileged status of the eye (202) [reviewed in (30)].

In contrast to pre-existing immunity, induction of the immune system after gene therapy in the eye was observed more frequently (203), albeit not to the same extent reported for other delivery routes (detailed in previous sections). Induction of a dose-dependent inflammatory response was observed in animal models (204–207) and in humans (208–212). In several clinical trials using either of the two delivery methods (subretinal or intravitreal), inflammation occurred but could be treated with immune suppression regimes. Likewise, transient antibody response against the capsid and cellular immune responses were also noted [reviewed in (30, 76, 213)]. The low to mild and transient immune responses allowed repeated administration of AAV vectors, either contralateral or in one eye (214, 215) or the delivery of two boluses in the same eye (216). Finally, long-term expression could be achieved in most clinical trials, with a decline observed in some after a few years (202, 210) [reviewed in (30, 76, 213)].

##### 3.1.2.3 Liver

Liver is the largest organ in the body, whose sinusoids filter an antigen-rich blood. In order to protect itself from the antigenic overload of nutritional components, chemicals and drugs, liver promotes immune tolerance rather than reaction (217). Even though systemically-delivered liver-targeted gene therapy cannot evade pre-existing immunity which challenges the immune-privileged status of the liver, the ability to harness the tolerogenic hepatic microenvironment has encouraged substantial research in the gene therapy field (145), especially regarding the expression of transgenes that are absent in subjects prior to the injection (187) [reviewed in (95, 175)]. Tolerance is dose-dependent, with higher dosage ensuring immune tolerance through Tregs, IL-10 expression, Fas-L and depletion of Teff cells as enabling factors (88, 110, 218). Another important consideration is whether the transgene product is secreted or not. Secreted proteins are presented in multiple organs and thus need a much lower threshold to produce Tregs. Interestingly, intracellularly restricted expression, e.g., using proteins located in the cytoplasm, results in antigen presentation by liver-draining lymph nodes (celiac and portal) and production of Tregs that are then disseminated to the periphery (96). This tolerance can be harnessed even with gene therapy targeted to other organs. Simultaneous expression of the transgene in the liver can induce expression of Tregs and tolerance to the transgene product expressed in muscle (104, 186). Most interestingly, the induction of immune tolerance through liver can be achieved despite the established presence of inhibitors (219) or even after gene delivery to another organ (88), hence reversing existing immunity [reviewed in (3, 30, 85, 138)].

#### 3.1.3 Induction of Immune Tolerance

Induction of immune tolerance can be achieved by targeting the liver, even at a later timepoint, as elaborated on in previous sections. Alternatively, Tregs can be *ex vivo* reprogrammed and adoptively transferred (97), or they can be induced through molecules (Tregitopes) or chemically (rapamycin). Tregitopes (Treg epitopes) are peptides found within human IgGs and exhibit high-affinity binding to MHCII. They were initially identified in humans (220) and are conserved in mammalian species (221). *Via* a mechanism requiring cell-to-cell contact, Tregitopes can trigger the proliferation of Tregs. Fusion of Tregitopes to the capsid proteins could also reduce CD8+ T-cell reactivity by fostering proliferation of Tregs *in vivo* (105). Alternatively, immunomodulatory drugs, such as rapamycin, can be used to induce Tregs. The phosphatidylinositol 3-kinase/protein kinase B/mammalian target of rapamycin (PI3K-Akt-mTOR) pathway regulates thymic and peripheral Treg generation (222). Rapamycin, an immunosuppressing compound used in graft rejection, can expand CD4+CD25+FoxP3+ Tregs (223). A hallmark study showed that simultaneous administration of synthetic vaccine particles encapsulating rapamycin [SVP(Rapa)] with AAV vectors alleviated anti-capsid humoral and cellular immune responses, thus enabling vector re-administration. Adoptive transfer of splenocytes to naïve mice transferred this immunomodulatory property while depletion of CD25+ cells neutralized this effect (89). Gene transfer to seropositive rhesus macaques after rapamycin treatment was only successful using subcutaneous, but not intravenous delivery (224), implying that route and dosing schedule may be key to success (225). Further studies in preclinical models are necessary to assess the efficacy of these treatments in the clinic as well as their safety, as they compromise the immune system and make the host vulnerable to infections [reviewed in (85, 138)].

#### 3.1.4 Immune Suppression

Immune modulation involves a broad range of approaches, of which the earliest and most widely applied in human clinical trials is transient immune suppression predominantly of T-cells with corticosteroids, such as prednisolone (188) [reviewed in (30, 85, 98, 226)]. Immune suppression can also be mediated using alternatives to steroids, such as mycophenolate mofetil (MMF) and tacrolimus (227), or MMF and rapamycin (98). Immune suppression has had varying effects in studies and clinical trials. Despite positive results (27, 101, 142, 228, 229), it often did not sustain long-term expression of the transgene (88, 230). Of note, sustained immune suppression with daclizumab is associated with a reduction in Tregs (111). Therefore, a recent study proposed new protocols using a meticulously timed T-cell-directed IS, with an early administration of MMF and rapamycin and a delayed delivery of anti-thymocyte globulin (ATG), to determine the balance between immunogenicity and tolerance (98). Immune suppression regimens were also used to deplete B-cells and thereby nAbs, such as rituximab with cyclosporine (231), rituximab with sirolimus (90), or rituximab alone (232), rapamycin without or with prednisolone (89, 233), and anti-CD20 with rapamycin (234). However, one should be aware that such regimens target the immune system of the host as a whole and are not specifically tailored to the gene therapy. Finally, another intriguing approach that has shown great promise but requires further studies is inhibition of proteasomal processing of internalized capsids, which is necessary for antigen presentation and part of the immune reaction process, using a variety of molecules (146, 148) some of which are also approved for use in humans (146) [reviewed in (138)].

#### 3.1.5 Depletion of Neutralizing Antibodies

Immune suppression offers only moderate capabilities to evade pre-existing immunity, which would otherwise disqualify 15-50% of the population from clinical trials. Accordingly, additional approaches are urgently needed (235). Recently, a hallmark study illustrated the potential of Imlifidase (IdeS), a cysteine endopeptidase derived from the immunoglobulin G (IgG)-degrading enzyme of *Streptococcus pyogenes*. IdeS cleaves human IgG into F(ab’)_2_ and Fc fragments, thus eliminating its Fc-dependent effector functions. In this study, the authors successfully showed the cleavage of human intravenous immunoglobulin (IVIg, a cocktail of serum from thousands of human individuals) *in vitro*, as well as in pre-immunized mice and seropositive NHPs *in vivo*. IdeS administration prior to AAV gene transfer *in vivo* reduces the levels of IgGs, thereby allowing efficient transduction. Treatment with IdeS also enabled readministration of AAVs. Finally, the efficiency of IdeS was also successfully validated with human sera (236). In a similar study, IdeZ, an IdeS homolog isolated from *S. equi* ssp. *zooepidemicus*, showed efficient IgG cleavage of dog, monkey and human sera and facilitated *in vivo* gene transfer in passively immunized mice and macaques (237). Moreover, a large protein, protein M, was identified in *Mycoplasma* that binds Igs with broad reactivity by using a different mechanism. *In vitro* and *in vivo* studies showed the efficiency of this approach to protect AAVs from nAbs and to allow readministration (235, 238).

Removal of anti-AAV nAbs has also been attempted through plasmapheresis, an extracorporeal method in which a device separates plasma from the cellular component of the blood. Afterwards, the plasma is filtered through various techniques, remixed with the blood cells and returned to the subject. Alternatively, the plasma can be substituted by a replacement solution. Plasmapheresis was used to remove immunoglobulins against the capsid in humans (239) and in NHPs (77). In the latter study, AAV gene transfer after plasmapheresis resulted in efficient transduction (77). While promising, this approach has several limitations including that multiple rounds are needed. A further concern is the so-called “antibody rebound” effect, which means that the antibody pool is readily replaced. Also, certain patients with weak physical conditions might be more adversely affected by the procedure, and the complete removal of immunoglobulins leaves the patients vulnerable to infections (77, 239) [reviewed in (47, 138)]. Recently, elegant studies have thus combined plasmapheresis with immunoadsorption columns loaded with empty AAV capsids, in order to selectively deplete nAbs against the AAV capsid (78, 79). In a similar manner, a prior study exploited empty AAV capsids that were injected to mice together with the AAV vectors to act as decoys for AAV-specific nAb (240). However, a concern is that excess AAV capsids could elicit stronger cellular immune responses or immunotoxicities (151) [reviewed in (42)]. It should also be noted that these procedures non-discriminately remove all AAV-binding antibodies, some of which do not neutralize but actually enhance transduction or alter distribution (126, 241).

### 3.2 AAV Vector Engineering

#### 3.2.1 Novel Serotype Isolation

The majority of the AAV serotypes that are currently in clinical use are human or NHP isolates from liver or spleen [reviewed in (1, 2)]. However, a concern with their use is that epidemiological studies show a high seroprevalence of those serotypes in the human population (114, 115, 121, 242, 243). A rational approach to overcome this concern is to isolate novel natural AAV serotypes from other species, which are expected to exhibit lower seroprevalence in humans but may also display lower transduction efficiencies. To this end, novel native AAVs have already been isolated from avian (244), rat and mouse (14), caprine (245), porcine (246, 247) and bat (80, 248) species. Some of these showed promise in *in vivo* biodistribution studies where they were found capable of targeting multiple tissues (246), such as heart (246), muscle (246, 247, 249), lung (245) or retina (247). Despite these promising results, further studies are necessary in larger, preclinical animal models to better characterize and ideally validate their potential for clinical application.

#### 3.2.2 AAV Capsid and Transgene Engineering

##### 3.2.2.1 Rational Capsid Design

There is a growing amount of information on immunogenic epitopes on the AAV capsid, based on cryo-EM structural mapping studies (250–255) [reviewed in (253)], single point mutants (256–258), or barcoded (9, 259) or not (260, 261) libraries of pooled vectors carrying point mutations. For the cryo-EM studies, monoclonal antibodies (mAbs) predominantly from hybridoma screens have been used, although recently, the isolation of human mAbs that are more clinically relevant has also been pursued (262). These studies facilitate the rational design of vector capsids or the engineering of libraries directed at the immunogenic epitopes, in order to render the complexity of the library technically attainable and to concurrently circumvent unwarranted changes to essential properties, such as tropism. Such information was applied to enhance nAb evasion by mutagenizing single [V719M (263), S671A (264), 265T (256)] or multiple positions (265, 266). A similar approach was used to modify surface tyrosines to avoid ubiquitination (267), proteasomal degradation and antigen presentation (149, 268, 269). This can be extended to other amino acids (serine, threonine, and lysine) (270, 271) and serotypes (272). Polyploid vectors, also called mosaic, combine capsid subunits from different serotypes and are formed by simply mixing the production plasmids at different ratios. This methodology identified a triploid AAV2/8/9 vector that could evade immunity more than the parental vectors (273).

A conceptually different approach is to display peptides on the surface of the capsid to evade or quench the immune system. Display on the AAV capsid of a self-peptide (SP), a 21-amino-acid long truncated bioactive form of CD47, whose binding to SIRPα (Signal regulatory protein α) on macrophages acts as a “don’t-eat-me” signal for macrophages, was shown to reduce macrophage uptake (91). Likewise, fusion of Tregitopes on the AAV capsid protein VP1 reduced CD8+ T-cell responses and increased Tregs (105).

##### 3.2.2.2 Rationally Designed Capsid Libraries and Directed Evolution

The wealth of knowledge regarding the immunogenic epitopes on the AAV capsid has been mostly implemented in rational engineering and directed evolution of viral libraries, to create novel, engineered immune evading AAVs. In an early study, five immunogenic amino acid positions (449, 458, 459, 493, 551) were randomized on AAV and the library was evolved under negative selection pressure using IVIg, producing immune-escaping variants (260). By studying the AAV1 complexed with four different Fabs of mouse anti-AAV1 mAbs, the cryo-reconstructed structures revealed three capsid antigenic footprints: region IV (456-AQNK-459), region V (492-TKTDNNNS-499), and region VIII (588-STDPATGDVH-597). Each region was separately randomized, and the libraries subjected to iterative rounds of evolution on vascular endothelial cells that are highly permissible to AAV1, in order to evolve the library for properties other than efficient transduction. The top variants were then combined albeit some could not be juxtaposed, as observed previously (274). In this case, the variant was combined with a library based on another footprint and a new evolution was applied. One of the evolved CAMs (Capsid Antigenic Motifs), CAM130, was significantly enhanced compared to the parent vector and, whilst maintaining tropism, showed an advantageous, immune-evading serological profile (81). A similar study evolved an AAV8-based variant, AAVhum.8, that exhibits mouse and human hepatocyte tropism and sera-evading properties (275). Another group harnessed a mAb PAV9.1 selected from a panel of hybridoma clones to identify a conformational epitope on AAV9 for further mutagenesis that comprises 494-TQNNN-498 and 586-SAQAQ-590. These Fab complementary determining regions (CDRs) were mutated using serotype swapping, alanine replacement, and additional point mutations. The resulting CDRs were efficient at evading the mAb binding, but they could not evade binding or neutralization by polyclonal serum or plasma from mice, macaques, or human donors (276). Another recent rational approach focused on residues identified to be different among 150 AAV3B variants, selected the surface-exposed ones and mutagenized them only to naturally occurring residues in this position. The library was evolved in human hepatocarcinoma spheroid cultures and the top variant, AAV3B-DE5, was further evaluated. It exhibited reduced seroreactivity against IVIg and individual human samples as well as tropism towards human, but not mouse hepatocytes in chimeric livers, similar to the parental serotype (277). Rational design strategies have contributed significantly to the field. However, screening of completely randomized libraries without *a priori* knowledge of immunogenic epitopes also offers valuable solutions, as discussed next.

##### 3.2.2.3 Randomized Capsid Libraries and Directed Evolution

Complementing the efforts outlined in the prior chapter that rely on limited antecedent knowledge to facilitate some degree of rational design followed by further AAV evolution, many groups have devised and applied experimental forward-oriented strategies in order to identify immunoevasive AAV variants. To this end, comprehensive libraries of synthetically engineered AAV capsid variants are first generated and then subjected to a negative selection pressure, which ideally eliminates all candidates that cross-react with, and are neutralized by, anti-AAV antibodies. The methodologies to create such diverse capsids libraries are manifold and have been extensively reviewed in the past by us and others (2, 278), hence it may suffice below to name some of the most widely used approaches including DNA family shuffling, peptide display, error-prone PCR and ancestral reconstruction.

One of the first studies to illustrate the power of directed AAV library screens and antibody-mediated selection was reported by the Kay lab in 2008 (279), which exploited the fact that IVIg contains a mixture of anti-AAV antibodies that is a good proxy for the human population. Accordingly, the group first created a library of ~7×10^5^ shuffled AAV capsid variants from eight parental viral serotypes and then iteratively amplified this capsid pool on human liver cells in the presence of IVIg, with the aim to eliminate all variants that were recognized by the anti-AAV antibodies. Indeed, this strategy enabled the isolation of a single chimeric AAV variant, called AAV-DJ, that at least partially resisted antibody neutralization *in vitro* and *in vivo* to a much greater extent than AAV2, which is one of its dominant parental serotypes and which has been used extensively in humans to date. The ability of shuffled AAV capsids to partially evade IVIg neutralization was later also confirmed by several other groups including notable work from Koerber et al., albeit this group only used IVIg during the stratification of already isolated chimeric AAVs and not for selection (280).

Similarly, in another representative example from the Schaffer lab (261), negative selection *via* a neutralizing anti-AAV2 rabbit serum was harnessed to enrich antibody-resistant AAV capsid variants from libraries that had been created through error-prone PCR amplification of the entire AAV2 *cap* gene. Interestingly, their lead candidate had not only become more antibody-resistant than wild-type AAV2 but had also acquired additional properties, such as altered DNA packaging efficiency, heparin affinity or cell specificity, likely explained by the well-known pleiotropic roles of many residues within the AAV capsid proteins.

These conclusions were confirmed in a flurry of more recent work from several labs, which cannot be covered comprehensively here; hence we will only highlight examples below that are representative for numerous other studies. One such example is another pivotal study by the Kay lab (82) in which Paulk and co-workers performed a multiplexed AAV library screen combining iterative *in vivo* selection of a shuffled library in “humanized” mice (*i.e*., mice xenotransplanted with human hepatocytes), followed by two rounds of *ex vivo* AAV capsid depletion on IVIg-coated beads. Of the shuffled capsid variants enriched by this procedure, NP59 is remarkable as it combines robust and specific *in vivo* transduction of human hepatocytes with good performance in seroreactivity and transduction neutralization assays, including the use of individual serum samples from macaques or humans (healthy or hemophilia B patients), or again IVIg. A very similar approach has also been reported more recently by the Li lab (281), using a different starting library and resulting in unique shuffled AAV variants. As a last example in this category, a study by the Samulski lab should be pointed out, which is remarkable for the fact that Li et al. selected a shuffled AAV capsid library in the presence of neutralizing sera not only in cultured cells, but directly in the muscle of mice (282, 283). Furthermore, rather than using an IVIg pool for selection, the group harnessed individual sera from human patients who had participated in a clinical trial for Duchenne muscular dystrophy with an AAV2.5 vector. A particularly notable conclusion in this work was such a stringent selection strategy may be beneficial, since pools such as IVIg are a mixture comprising sera without neutralizing anti-AAV antibodies, hence AAV capsid variants emerging from IVIg selection may only escape neutralization in subjects with high individual antibody titers, whereas those selected with a stringent serum may be more broadly resistant.

Similar conclusions were also drawn in parallel work in which capsids were diversified not *via* DNA shuffling, but rather *via* insertion of short peptides (typically 7 to 14 amino acids long) on the capsid surface. While the primary purpose of these peptides is binding to (usually unknown) receptors on target cells, several groups have reported that display of these short additional peptides on the AAV shell can also modulate its recognition by neutralizing antibodies. As a representative example, one of the first studies reporting this finding should be highlighted (284), in which Huttner and colleagues found that insertion of peptides selected in previous screens in AAV2 amino acid positions 534 and 573 substantially reduced capsid affinity for neutralizing anti-AAV2 antibodies in human sera.

In general, it should be an interesting goal for future work to compare the lead candidates from these and other studies side-by-side in genuine or humanized mouse livers in the presence of anti-AAV antibodies, ideally using a vector DNA/RNA barcoding strategy (285) to enable a fair comparative analysis in the same animal(s). Besides, the aforementioned research, along with the rational design studies, further corroborates the notion that antigenic, tropism and potency determinants overlap in the structural context of the AAV capsid and thus perfectly complements the alternative strategies noted above, not only on a technical but also on the biological level.

##### 3.2.2.4 Chemical Capsid Modifications

Rather than engineering the capsid itself, either *via* directed evolution or rational design, numerous groups have pursued an alternative and complementary strategy to mask AAV from neutralizing antibodies, by either chemically modifying the shell or encapsulating the particles in extracellular vesicles (exosome). As with the capsid engineering approaches, the diversity of strategies is so substantial and the literature so complex that we can only highlight a few representative examples below, and we apologize to all colleagues whose work we had to omit for space reasons.

One particular strategy that has been reported frequently is AAV modification *via* chemical conjugation with polyethylene glycol (PEG), which is a simple, cheap and effective means to cover and protect the capsid from neutralization, but which may also come at the cost of (steric) interference with AAV transduction and tropism. This dilemma was exemplified, for instance, in one of the first reports of AAV PEGylation by Lee and co-workers (286) who found that there is only a small window of PEGylation, *i.e.*, PEG:lysine conjugation ratio and PEG molecular weight, that enables effective antibody protection while maintaining infectivity. Subsequently, other used various alternative strategies for AAV PEGylation, such as AAV2 modification with a series of activated PEGs, some of which yielded protection from neutralization without severely impeding transduction efficiency (287), or use of genetic code expansion for insertion of a lysine mimic into the AAV2 capsid that enabled site-specific PEGylation and escape from neutralization (288).

Instead of coating the AAV particle *via* chemical modification, other groups, most notably the one of Casey Maguire, rather encapsulate the vector particles in naturally occurring cellular vesicles, resulting in what was originally called “vexosomes” and later exo-AAVs. As, for instance, demonstrated by György et al. in 2014 (83), exosome-encapsulated AAV9 vectors were significantly more resistant to both, pooled human serum as well as IVIg, and they also performed better than naked AAV9 in passively IVIg-immunized mice. Importantly, this method is not restricted to a particular serotype, since escape from neutralization in cultured cells was also observed for AAV1 and AAV2. These encouraging results were corroborated and extended in a series of more recent studies, including a notable piece of work from Meliani et al. who showed that exo-AAV8 vectors (and also exo-AAV5) performed better than the wild-type counterpart at liver-directed human factor IX expression in mice, perhaps owing to a faster nuclear translocation rate and autophagy-independent trafficking. Remarkably, in turn, the higher expression was also correlated with an increased frequency of Tregs in lymph nodes of exo-AAV8-treated mice, suggesting that improved induction of immunological tolerance may be an additional benefit of exosome-encapsulated AAVs. Moreover, exo-AAV8 was also more resistant to neutralizing anti-AAV8 antibodies in human sera and in a passive immunization mouse model of liver gene transfer, further illustrating the potential of this strategy to expand the proportion of human subjects who are eligible for AAV gene therapies.

Finally, another intriguing approach worth mentioning has recently been reported by Katrekar and colleagues (289), who combined genetic code expansion and click-labeling to precisely tether oligonucleotides to the surface of the chimeric AAV-DJ capsid (279). When incubated with lipofectamine, this resulted in so-called “cloaked” AAVs that were much more resistant to neutralizing anti-AAV antibodies and concurrently yielded higher CRISPR gene editing efficiencies than an unconjugated AAV-DJ control, reminiscent of the dual benefit observed with the exo-AAV strategy (see above).

While these and other, equally compelling studies that could unfortunately not be mentioned here are very promising, it is also clear that additional, meticulous work is needed and that a number of challenges have to be overcome before chemically modified AAVs can be used more broadly and even clinically. This includes the need to optimize and standardize large-scale production and purification protocols, as well as the requirement of a thorough characterization of possible impurities and contaminations especially in cell-derived exo-AAVs. In addition, immune responses against PEG or lipofectamine will have to be studied and, if detected, may limit the widespread application of certain formulations. Last but not least, cloaking or coating the viral shell may inadvertently and negatively impact *in vivo* features such as biodistribution, kinetics and blood clearance, which would be particularly detrimental for synthetic capsids that have been genetically engineered to have a more defined tropism or other advantageous properties.

##### 3.2.2.5 Vector Genome Optimization

Next to the viral capsid, also the cargo, *i.e*., the recombinant genome consisting of the transgene expression cassette flanked by the ITRs, offers multiple opportunities for engineering and alleviation of immune responses, including its structure and components.

Regarding structure, a favorable design with respect to efficiency are self-complementary (sc) or double-stranded AAV vector genomes, which―after replication and encapsidation―carry two inverted copies of a transgene that rapidly and effectively self-anneal in the transduced cell. Thereby, scAAV genomes alleviate the rate-limiting step of second-strand DNA conversion that normally restricts transduction with conventional single-stranded AAV vectors and that explains their slow kinetics of transgene expression that is typically observed *in vivo*. While beneficial in this aspect, a drawback of scAAV vector genomes that has become apparent over the last decade is their higher propensity to trigger an innate immune response *via* the endosomal DNA receptor TLR9, as observed in several tissues including the liver and the muscle. This includes early data in mice where scAAV vectors led to increased expression of various innate immune-related genes and induced innate responses in a dose-dependent manner *via* TLR9 signaling. This, in turn, enhanced adaptive immune responses to the capsid but not the transgene product, probably due to the short-lived and self-limiting nature of the innate response (58).

Still, in independent work, the Ertl lab showed that scAAV vectors, as a result of their faster transgene expression kinetics, are also more prone than ssAAV to induce transgene product-specific CD8+ T-cell and B-cell/antibody responses in mice (164). The extent of these responses depended on the capsid serotype, with AAV7 yielding much stronger effects than AAV2, probably owing to the higher efficiency of AAV7 in the muscle.

While both studies unanimously concluded that AAV genome configuration governs the immunogenicity of AAV particles, the authors also concurred that lowering doses may allow scAAV vectors to dodge the immune system. Alternatively, or in addition, as demonstrated consistently by the Wilson, Ertl and Church labs, innate immune responses can also be blunted by directly engineering the vector genome. First, Faust and colleagues reported that vector genomes depleted of CpG islands, which are typically sensed by TLR9, evade the innate and adaptive immune response and establish persistent transgene expression in skeletal muscle in mice in the absence of T-cell infiltrates, even when delivered by a highly immunogenic AAVrh32.33 capsid (67). Concurrent with this, and as already noted above in chapter 2.2.2.1, the Ertl lab found that CpG depletion in AAV vector genomes can diminish a primary, *de novo* T-cell response, by reducing expansion of naïve CD8+ T-cells against AAV capsid epitopes (63). In contrast, these engineered vectors triggered a secondary response, by driving proliferation of anti-AAV capsid-specific memory CD8+ T-cells, a phenomenon that was also observed with empty AAV capsids. The latter is particularly relevant in view of the fact that spiking in empty capsids into AAV vector preparations has previously been shown to dampen the humoral immune response to the viral particles, implying that the empty capsids acted as antibody sponges. Hence, the findings by Xiang et al. (63), that empty capsids do not stimulate a primary T-cell response to antigenic capsid epitopes and also do not boost T-cell activation triggered by full AAV particles, are pivotal for our understanding of the role of full *versus* empty capsids and for the future optimization of strategies to circumvent humoral and cellular anti-AAV immune responses.

Most recently, Chan and co-workers moreover showed that TLR9 activation can also be dampened through incorporation of short oligonucleotides that antagonize TLR9 activation, dubbed TLR9i (i for inhibitory) (86). Such TLR9i sequences were found to cloak the AAV vectors in multiple, but not all tested animal models and tissues, confirming the pivotal role of TLR9 sensing but concurrently implying the existence of other, TLR9-independent immune mechanisms. The latter may also have contributed to the results of clinical trials using AAV8 for expression of the human blood coagulation factor IX in hemophilia B patients, in which capsid-specific CD8+ T-cell responses were observed despite the use of CpG-reduced vector genomes (101). In this context, another original hypothesis that is noteworthy and that was presented by Li and Samulski (1) suggests that the intrinsic promoter activity of the AAV ITRs might drive the generation of dsRNA at later stages of transduction, in turn activating cellular RNA sensors and thus stimulating an innate immune response. If true, this would imply a solution whereby this promoter activity is diminished or blocked, by deliberately engineering the ITRs and/or insulating the expression cassette from the ITRs (1).

Interestingly, the authors of the latest work on TLR9i postulated that these sequences may act by outcompeting CpG islands in the vector genome for TLR9 binding, potentially *via* their higher TLR9 affinity and their ability to prevent TLR9 dimerization and activation upon binding (86). While not tested in this work, this raises the intriguing question whether combining TLR9i with the aforementioned depletion of CpG islands may synergize and further dampen innate immune responses to the AAV vector genome. Additional topics for future work should include the persistence of the effect and the impact on vector transgene expression, as well as on clinically relevant immune responses, including humoral or cellular immunity to the capsid proteins or the transgene product. Furthermore, it will be prudent and informative to assess this strategy in more animal models and with other targets, using different clinically applicable administration routes. Until then, the fact that Chan et al. noted beneficial effects on the anti-AAV immune response in a variety of experimental settings and largely independent of the other vector components, such as capsid or promoter, is already highly encouraging as it suggests a large degree of versatility, modularity and translatability.

## 4 Toxicity

Despite the success of AAV-based gene therapeutics and the prevailing view that AAV is less immunogenic than other recombinant virus platforms, rapidly mounting evidence from preclinical work in large animals and clinical studies in humans implies that AAV vectors can cause inflammatory and immune responses as well as other dose-dependent toxicities and pathologies. This includes observations of severe adverse events, possibly related to innate and cellular immune responses, in patients suffering from Duchenne muscular dystrophy (DMD) or spinal muscular atrophy type 1 who had been treated with high AAV vector doses (290, 291). In the DMD trial (IGNITE), treatment of a boy with a high dose (2x10^14^ vg/kg) of the therapeutic vector (SGT-001, encoding micro-dystrophin) resulted in lower red blood cell and platelet counts (thrombocytopenia), caused kidney damage, and activated the complement system. Fortunately, these complications were all resolved and the sponsoring company (SOLID) improved their manufacturing protocol (to reduce the number of empty capsids), eventually allowing the FDA to lift their initial ban on this trial.

Similar findings were reported in a phase I trial (sponsored by Rocket Pharmaceuticals) for treatment of Danon disease, a devastating and rare X-linked autophagic vacuolar myopathy that results from mutations in the LAMP-2 gene and that can cause dysfunction of the musculature and other organs, frequently triggering early mortality. In this trial, one patient treated with the high dose (1x10^14^ vg/kg) and who had a pre-existing anti-AAV9 immunity, experienced adverse events that were presumably immune-related and that also comprised thrombocytopenia and acute kidney injury. Luckily, also this patient ultimately recovered and regained normal organ function. Still, the serious and consistent adverse events in these two trials clearly raise a warning flag about possible toxicity from *in vivo* application of excessive AAV vector doses.

In addition, AAV dose-dependent pathology was observed in dorsal root ganglia (DRG) in vector-treated non-human primates that seemed to be largely independent of capsid or cargo (292, 293). The effects were only mild to moderate and not associated with fatalities; moreover, a possible mitigation has recently been proposed in which the inclusion of binding sites for miR-183 a miRNA largely restricted to DRG neurons, can alleviate DRG toxicity (294). Nonetheless, the possible link between AAV delivery and sensory neuropathies in DRGs requires further investigation and careful monitoring in ongoing or future clinical studies.

However, most dire and most alarming is the outcome of the recent ASPIRO trial, in which three children affected by X-linked myotubular myopathy (XLMTM) and given a very high dose of 3x10^14^ AAV8 vg/kg bodyweight developed progressive liver dysfunction, bacterial infection and sepsis (two of the three patients), eventually resulting in death of all three individuals (295–298). Also here, a critical role of the immune system has been suspected, including the presence of anti-AAV antibodies in these patients that could have triggered an innate response or activated the classical arm of the complement system. Notably, none of the patients in this trial who had received a lower vector dose developed liver-related adverse events. Instead, several children regained the ability to sit, stand or walk and no longer needed ventilator support, clearly illustrating the great potential of this gene therapy approach and of the vector used. Of note is that no such toxicities had previously been observed with the same vector in mice or non-human primates, even using higher doses of 8x10^14^ AAV8 vg/kg, and that very encouraging efficacy data had been obtained in murine and canine XLMTM models (299, 300). This highlights the species-specific differences in AAV-host interactions and the urgent need for better characterization including a possible contribution by immune mechanisms, with the clinically highly relevant aim to control and overcome, or ideally altogether prevent, such adverse events in patients.

Last but not least, we note another very recent, serious adverse event in an AAV gene therapy clinical trial (INFINITY, company sponsor Adverum), this time the loss of sight in a patient with diabetic macular edema who was treated with a high dose (6x10^11^ vg/eye) of a vector based on a synthetic AAV capsid (AAV2.7m8). The fact that the eye is more immunoprivileged than other organs (see also chapter 3.12.2.) and that the effect occurred long (30 weeks) after dosing may argue at least against an acute anti-AAV immune response, but the mechanisms are still unclear.

## 5 Conclusions

The historic fable “The race between the hare and the hedgehog”, originally published by the Brothers Grimm in 1843, describes the race of two animals, one fast (hare) and one slow (hedgehog), of which the latter has no chance of winning the contest. Still, the hedgehog seemingly outcompetes the hare through cheating, as he places his lookalike wife at the finish line and thereby ultimately frustrates the hare to an extent that it dies. For many reasons, one can readily apply the same image and draw comparisons to the incessant arms race between AAV and its human host, starting with natural infections and nowadays significantly accelerated by the growing clinical use of recombinant AAV vectors. Akin to the fable, there is substantial hope that the hedgehog in this analogy, *i.e*., the AAVs, will eventually win this race against the host (the hare), assisted by a little cheating in the form of our ever expanding understanding of AAV-host interactions and the concurrent advent of powerful new technologies to blunt or alleviate anti-AAV immune responses, a collection of which has been highlighted and discussed in this article. It is also evident that this looming happy ending requires a considerable body of additional, concerted work from the field, aimed at a better characterization of the new, genetically engineered, evolved or designed, and/or chemically modified AAV particles along with improvements in the technology for their clinical-grade manufacturing including production, purification, quality control and batch release. Still, in view of the remarkable pace which the race between AAV and the host has picked up in recent years, there is every reason to hope and believe that the fairy tale of AAV gene therapies in the absence of adverse immune responses will eventually become a reality.

## Author Contributions

All authors listed have made a substantial, direct, and intellectual contribution to the work and approved it for publication.

## Funding

KR was supported by a Marie Curie International Incoming Fellowship PIIF-GA-2013-627329. DG greatly appreciates support by the German Research Foundation (DFG) through the DFG Collaborative Research Centers SFB1129 (Projektnummer 240245660) and TRR179 (Projektnummer 272983813), as well as by the German Center for Infection Research (DZIF, BMBF; TTU-HIV 04.819). DG is moreover grateful for support from the ANR/DFG-funded project MATRIXNASH and the DLR-funded project KARTLE.

## Conflict of Interest

DG is a co-founder and shareholder (CSO) of AaviGen GmbH. DG and KR are inventors on a pending patent application related to the generation of immune-evading AAV capsid variants.

## Publisher’s Note

All claims expressed in this article are solely those of the authors and do not necessarily represent those of their affiliated organizations, or those of the publisher, the editors and the reviewers. Any product that may be evaluated in this article, or claim that may be made by its manufacturer, is not guaranteed or endorsed by the publisher.
